# All-Trans Retinoic Acid-Induced Cell Surface Heat Shock Protein 90 Mediates Tau Protein Internalization and Degradation in Human Microglia

**DOI:** 10.1007/s12035-024-04295-1

**Published:** 2024-06-20

**Authors:** Ngoc Lan Nguyen, Thi Xoan Hoang, Jae Young Kim

**Affiliations:** Department of Life Science, Gachon University, Kyeonggi-Do 13120, Seongnam, Korea

**Keywords:** All-trans retinoic acid, Tau protein, Cell surface heat shock protein 90, Microglia, Tau uptake

## Abstract

This study investigates the role of all-trans retinoic acid (ATRA) in modulating the expression of heat shock protein 90 (Hsp90) and its influence on the uptake and degradation of tau proteins in immortalized human microglia cells. We demonstrate that ATRA significantly upregulates Hsp90 expression in a concentration-dependent manner, enhancing both extracellular and intracellular Hsp90 levels. Our results show that ATRA-treated cells exhibit increased tau protein uptake via caveolae/raft-dependent endocytosis pathways. This uptake is mediated by surface Hsp90, as evidenced by the inhibition of tau internalization using an extracellular Hsp90-selective inhibitor. Further, we establish that the exogenously added full-sized monomeric tau proteins bind to Hsp90. The study also reveals that ATRA-enhanced tau uptake is followed by effective degradation through both lysosomal and proteasomal pathways. We observed a significant reduction in intracellular tau levels in ATRA-treated cells, which was reversed by lysosome or proteasome inhibitors, suggesting the involvement of both degradation pathways. Our findings highlight the potential therapeutic role of ATRA in Alzheimer’s disease and related tauopathies. By enhancing Hsp90 expression and facilitating tau degradation, ATRA could contribute to the clearance of pathological tau proteins, offering a promising strategy for mitigating neurodegeneration. This research underscores the need for further exploration into the molecular mechanisms of tau protein internalization and degradation, which could provide valuable insights into the treatment of neurodegenerative diseases.

**Supplementary Information:**

The online version contains supplementary material available at 10.1007/s12035-024-04295-1.

## Introduction

Tauopathy plays a crucial role in various neurodegenerative diseases, prominently in Alzheimer’s disease (AD), a major cause of dementia [1]. Tau proteins, microtubule-associated proteins expressed mainly in neurons of the central nervous system (CNS), stabilize microtubule structures under normal conditions, especially in axonal neurons. Pathologically, phosphorylated tau reduces microtubule-binding affinity, leading to neuronal damage [2]. Not only are hyperphosphorylated tau and neurofibrillary tangles (NFT) toxic, but soluble forms like monomeric and oligomeric tau also detrimentally affect neurons and brain cells [3, 4]. Intracellular soluble tau can be secreted, posing toxicity to neighboring cells, including neurons and glial cells [5]. Tau propagation, similar to “prion-like propagation,” involves pathological tau seeds transferring from donor to recipient neurons, where they initiate new pathological tau aggregates [6, 7]. Thus, targeting toxic extracellular monomeric and oligomeric tau in the CNS is essential.

Tau interacts with heat shock protein 90 (Hsp90), which recognizes tau and modulates its conformation by binding to specific Hsp90 domains [8]. Hsp90, an ATP-dependent molecular chaperone, is vital for various cellular processes, including protein folding, assembly, stabilization, and degradation [9]. It also plays a key role in proteostasis during stress and the uptake of exogenous proteins [10, 11]. Hsp90 is localized not only within the intracellular matrix but also on the cell surface and in the extracellular milieu. In macrophages, cell surface Hsp90 plays a critical role in pattern recognition [12] and in the internalization of bacterial flagellin [10], thereby activating immune responses and defending against exogenous pathogens. While Hsp90’s role in tau research, particularly in aggregation and clearance, is documented [13], its exact function in tau internalization remains unclear. However, Hsp90 might act as a cell surface receptor facilitating tau uptake in various cells, necessitating further research to understand its role in tau internalization and related pathologies.

Microglia, macrophage-like cells in CNS, are integral to the brain’s innate immune system and play a significant role in neurodegenerative diseases [14]. Studies indicate involvement of microglia in tau pathology, including phagocytosis, expression, and clearance. However, microglial dysfunction can impede free tau degradation, escalating inflammatory responses and contributing to tauopathy [15]. Enhancing microglial function could be a potential therapeutic strategy for removing pathological extracellular tau.

Vitamin A, particularly its active form, retinoic acid (RA), is crucial for vision, immune system functions, and cellular growth and development. Among its forms, all-trans retinoic acid (ATRA) is the most prevalent in cells [16]. Vitamin A deficiency in the CNS has been linked to cognitive diseases, including Alzheimer’s and Parkinson’s diseases, and autism spectrum disorder [17, 18]. The specific impact of ATRA on tau pathology, however, remains underexplored.

In this study, we demonstrate that ATRA promotes the uptake and degradation of extracellular tau proteins by HMO6 cells via cell surface Hsp90.

## Materials and Methods

### Reagents and Cell Culture

We utilized the following chemicals: All-trans retinoic acid (Cat # R2625),methyl-beta-cyclodextrin (Cat # C-4555), Filipin III (Cat # F-4767), and leupeptin lysosome inhibitor (Cat # L2884) from Sigma-Aldrich (St. Louis, MO, USA); recombinant human tau protein (hTau42) (Tau-441) (Cat # SP-495-100) and human tau antibody (MAB10629-100) from R&D Systems (Minneapolis, MN, USA); FITC-Tau (Lot # 102020T441F) from rPeptide (Georgia, USA); biotinylated geldanamycin (GeB) from Invivogen ( Cat # ant-bgl-1) (San Diego, CA, USA); Dyngo®4a (ab120689), Alexa Fluor 647 anti-Hsp90 antibody (ab223468), and Epoxomicin proteasome inhibitor (ab144598) from Abcam (Cambridge, UK); Hsp90 antibody (sc-69703) from Santa Cruz Biotechnology (Dallas, TX, USA). DMEM (LM 001-01), DPBS (LB 001-01), and FBS (S001-01) were sourced from Welgene Inc. (Paris, France).

The HMO6 human immortalized microglia cell line (accession number CVCL_5G94) [19] was cultured in DMEM supplemented with 10% heat-inactivated fetal bovine serum (FBS), 100 µg/ml penicillin, and 100 µg/ml streptomycin (Invitrogen) (Cat# 15140122) at 37 °C in a 5% CO2 atmosphere.

### RNA Preparation and Quantitative Real-Time PCR (qRT-PCR)

Post-treatment, total RNA was extracted using the easy-BLUETM Total RNA Extraction Kit (Cat#17061) (iNtRON Biotechnology, Inc.) and quantified with the MaestroNano MicroVolume Spectrophotometer (Maestrogen, Las Vegas, NV, USA). cDNA was synthesized from 2 µg of total RNA using HyperscriptTM 2X RT Master Mix (Cat#601-710) (Geneall Biotechnology). Real-time PCR for targeted genes was conducted using EzAMPTM FAST One-Step RT-qPCR 2X Master Mix SYBR (Elpis-Biotech) (Cat# on a LineGene 9600 Plus Fluorescent Quantitative Detection System (Bioer Technology). mRNA expression was normalized to GAPDH as a control. Primers used were Hsp90α 5′-ACCTGGAGATAAACCCTGACCA-3′ and 5′-AGGAGCGCAGTTTCATAAAGCA-3′; GAPDH 5′-ACAGCCTCAAGATCATCAGCAAT-3′ and 5′-AGGAAATGAGCTTGACAAAGTGG-3′

### Flow Cytometry

Cells were detached and washed with DPBS 2 times. For cell surface protein analysis, after fixation with 4% formaldehyde for 10 min, cells were directly incubated with primary antibodies (1:50), followed by PE-conjugated secondary antibodies (1: 300) at 4 °C for 30 min. To detect intracellular proteins, cells were fixed with 4% formaldehyde for 10 min then permeabilized with 0.1% Triton-X for 10 min before antibody incubation. Cells were then resuspended in 400 µl DPBS for analysis using the Cytomics FC500 MLP (Beckman Coulter Inc., Fullerton, CA, USA).

To detect tau uptake, cells were fixed and permeabilized, then incubated with a human tau antibody for 30 min followed by a secondary antibody for an additional 30 min at 4°C. The level of tau internalization was measured by flow cytometry.

### Immunofluorescence

For detection of cell surface Hsp90, cells were fixed with 4% formaldehyde for 10 min then washed 2 times with DPBS while to measure intracellular Hsp90, after fixation, cells were permeabilized with 0.1% Triton-X at 10 min. Next, samples were incubated with primary antibody (1:100) at 4 °C overnight followed by FITC-conjugated secondary antibodies (1:1000) at room temperature for 30 min. For tau internalization detection, cells treated with FITC-tau under different conditions, then cells were washed twice with DPBS before fixing with 4% formaldehyde for 10 min. Nuclei were stained with Hoechst 33342 (1:1000) for 15 min. Fluorescence microscopy was performed using an Olympus CKX53 microscope (Tokyo, Japan) while signal of tau internalization were examined with a confocal microscope (Nikon, Japan).

### Western Blot

Cells were lysed in a 1% Triton-X (Cat#H5141) (Biocompare) and protease inhibitor (Cat# ab65621) (Abcam) mixture. Protein concentrations were determined using the Bradford Assay Kit (Bio-Rad). Proteins (20 µg) were separated on 7.5% SDS-PAGE and transferred to PVDF membranes (Cat#162177) (Bio-rad). Membranes were blocked with 5% BSA in TBST, incubated with primary antibodies overnight at 4 °C, then with secondary antibodies for 30 min. Detection was performed using ECL solution (Cat#W3652-020) (GenDEPOT) and a ChemiDoc MP system (Bio-Rad).

For the detection of secreted Hsp90 proteins, the culture medium was collected after ATRA treatment and then concentrated using an Amicon Ultra-15 centrifugal filter unit (Sigma-Aldrich #UFC901008). The concentrated proteins were subsequently analyzed by Western blot.

### Co-Immunoprecipitation (Co-IP)

Co-IP was conducted using the Pierce Co-IP Kit (Cat#26149) (Thermo Fisher). Lysates were prepared as in the Western blot protocol. For pre-clear step, cell lysis (1 mg) was incubated in a column pre-cleared with control agarose resin slurry for 30 min at 4 °C with a rotator. The pre-cleared lysates were then incubated on the column pre-coupled with anti-Hsp90 antibody at 4 °C overnight. Mouse IgG (Cat#10400C) (Thermo Fisher) was used as a negative control. The mixture was then collected by centrifugation at 1000 x g for 1 min. Post-centrifugation, the supernatant was reserved for unbound protein analysis, and the protein-bound bead pellet was washed and eluted for Western blot evaluation.

### Biotinylation Assay

A general procedure for the biotinylation assay is shown in Fig. 4a. Followed by treatment with ATRA or GeB + ATRA, cells were washed with ice-cold PBS and then incubated with 0.25 mg/mL Sulfo-NHS-SS-Biotin (Cat#A44390) (Thermo Fisher) in 48 mL ice-cold PBS on a rocking platform for 30 min at 4 °C Subsequently, the cells were washed twice with ice-cold PBS containing 20 mM glycine to remove the non-reactive biotin, followed by treatment with 10 ng/mL of human recombinant tau protein and incubated at 4 °C (as a negative control) or 37 °C (to allow the internalization) for 6 h before subjected to the following steps:

For the isolation of cell surface biotinylated proteins, the Pierce® Cell Surface Protein Isolation Kit (Cat#A44390) (Thermo Fisher) was used. Briefly, after incubation step, cells were washed twice with ice-cold PBS and lysed in 1% Triton-X containing lysis buffer. The cell lysis was collected, mixed with resin and subjected to a column pre-incubated with NeutrAvidin agarose and incubated at room temperature for 30 min in an end-over-end rotator. The cell surface biotinylated protein was then eluted using the elution buffer containing 10 mM DTT. The concentration of isolated protein was then determined by Bradford Assay Kit, and 20 µg of protein was used for the western blot analysis.

For internalized biotinylated protein, after incubation time at 37 °C, cells were placed on ice, washed twice with ice-cold PBS, and incubated for 30 min with the ice-cold GSH quenching buffer (containing 50 mM glutathione, 75 mM NaOH, 75 mM NaCl, 1 mM EDTA, 0.1% BSA). The cells were then washed twice with ice-cold PBS and subjected to a lysis procedure as described above.

### Enzyme-Linked Immunosorbent Assay (ELISA)

Human tau protein levels in culture supernatants were measured using indirect ELISA with modifications from [20]. Supernatants were added to high-binding plates overnight at 4 °C. After washing and blocking, samples were incubated with primary antibodies overnight at 4 °C, then with HRP-conjugated secondary antibodies for 1 h at room temperature. Post-wash, TMB substrate solution was added for 30 min, and the reaction was stopped using ELISA stop solution. Absorbance was measured at 450 nm using an ELISA Reader (µ-Quant, Bio-Terk Instruments, Winooski, USA).

### Statistical Analysis

Experiments were replicated at least three times. Data are presented as mean ± standard deviation (SD). Group differences were assessed using one-way ANOVA followed by post hoc tests in SPSS 12.0 for Windows, with significance set at *p* < 0.05.

## Results

### ATRA Upregulates Hsp90 Expression in HMO6 Cells

**Fig. 1:**
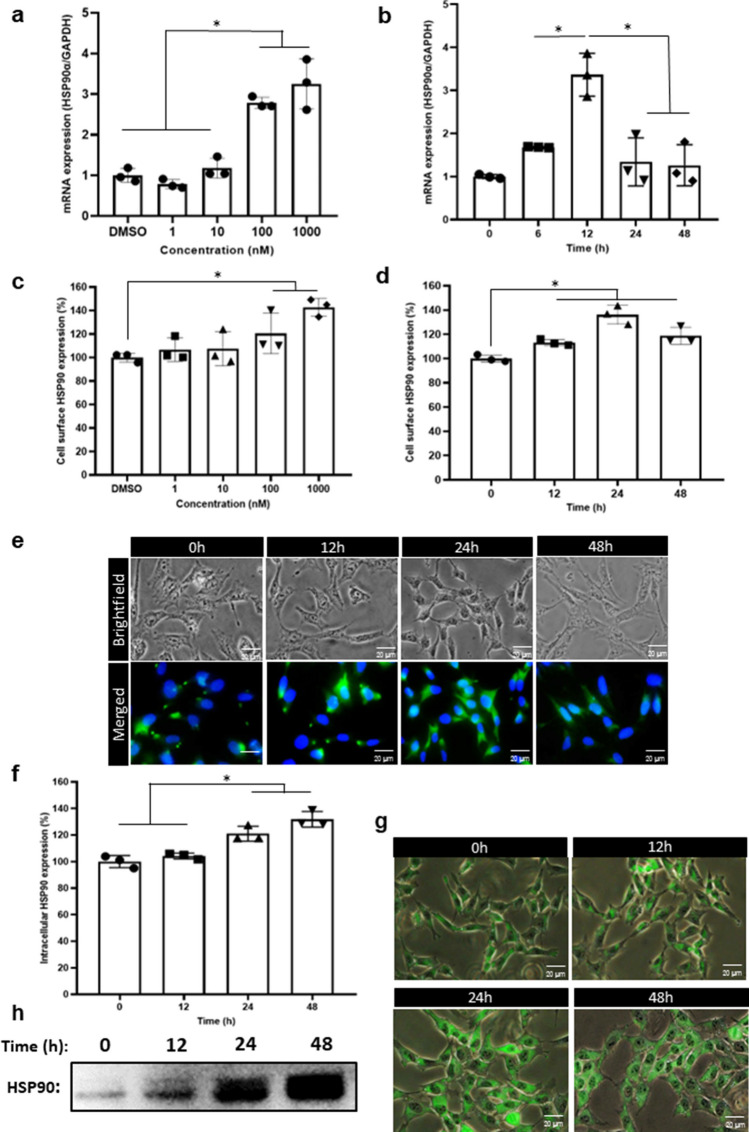
ATRA induces increased expression of Hsp90 in HMO6 cells. **a**–**b** mRNA levels of Hsp90 in HMO6 cells. Treatment of HMO6 cells with varying concentrations of ATRA for 12 h (**a**) or with 1 µM ATRA across different durations (**b**) was followed by evaluation of Hsp90 mRNA via qRT-PCR. **c**–**e** Cell surface expression of Hsp90 in HMO6 cells. Cells underwent treatment with either DMSO or varying concentrations of ATRA for 24 h (**c**), or with 1 µM ATRA over different time periods (**d**, **e**). Detection of Hsp90 on the cell surface was conducted using flow cytometry (**c**, **d**) and fluorescence microscopy (**e**, magnification ×200) (blue: nucleus (Hoechst); green: Hsp90 (FITC)). **f**–**g** Intracellular Hsp90 concentrations in HMO6 cells. Following treatment with 1 µM ATRA for various durations, intracellular Hsp90 levels were quantified using flow cytometry (**f**) and fluorescence microscopy (**g**, magnification ×200) (blue: nucleus (Hoechst); green: Hsp90 (FITC)). **h** Extracellular Hsp90 levels in HMO6 cells. Post-treatment with 1 µM ATRA at different time points, the culture medium was harvested and concentrated using an Amicon Ultra-15 Centrifugal Filter Unit (10 kDa). Subsequently, the levels of secreted Hsp90 were analyzed via Western blotting. *p < 0.05

Since ATRA enhanced cell surface Hsp90 expression in human macrophage cells in our previous study [10], we initially investigated the effect of ATRA on Hsp90 mRNA and protein levels in HMO6 cells. Cells were treated with DMSO or varying concentrations of ATRA for 12 h and 24 h, after which mRNA and protein expression levels were evaluated. Figure 1 shows that ATRA significantly increased Hsp90 mRNA in a concentration-dependent manner (Fig. 1a) and 12h treatment enhanced the highest level of gene expression (Fig. 1b). Flow cytometry and immunofluorescence results consistently indicated an enhancement in the levels of extracellular Hsp90 (Fig. 1c, d, e) and intracellular Hsp90 expression (Fig. 1f and g), with the treatment of 1 µM ATRA for 24 h inducing the highest level of cell surface Hsp90 (142.9%+/−7.5%). Therefore, we selected a 24-h treatment with 1 µM ATRA for subsequent experiments. Additionally, we assessed the level of secreted Hsp90 in the culture medium after various treatment durations with 1 µM ATRA using Western blot analysis. Figure 1h shows that the group treated with ATRA for 48h had the highest increase in Hsp90 secretion compared to the control group.

### ATRA-Enhanced Cell Surface Hsp90 Mediates Tau Uptake in HMO6 Cells

**Fig. 2:**
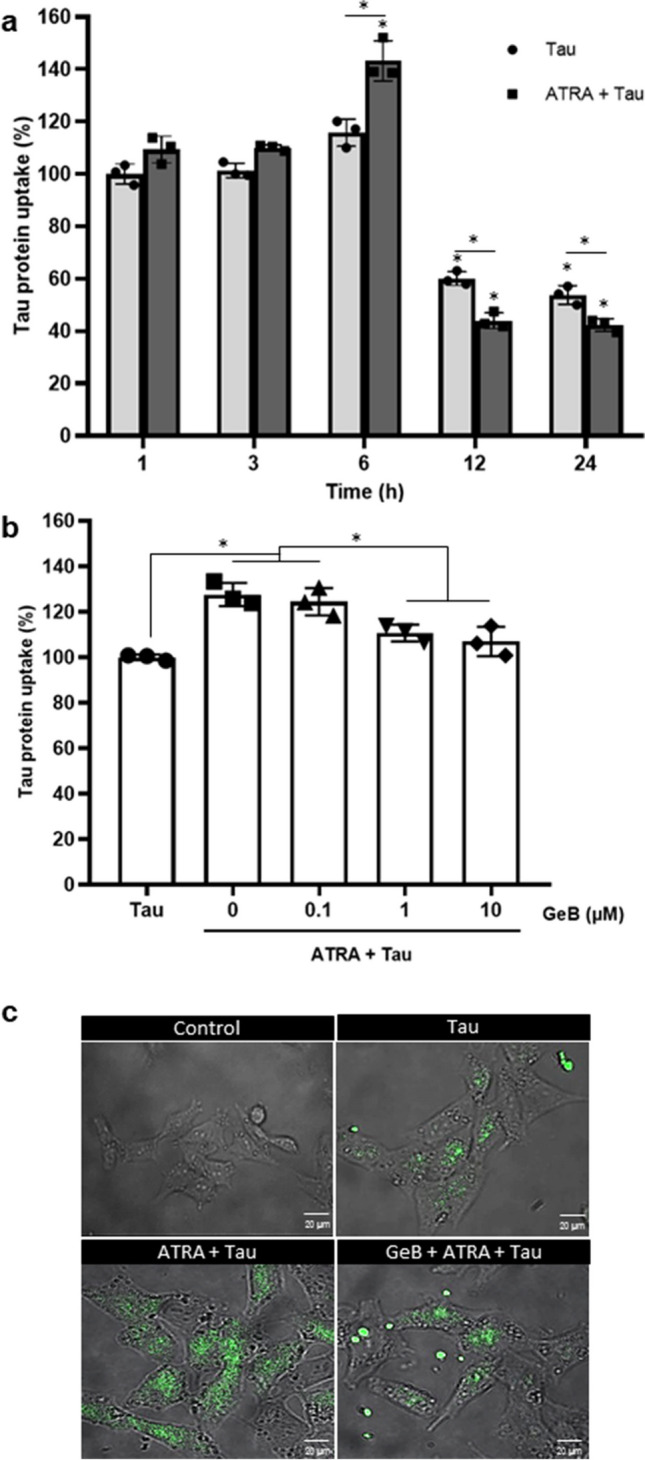
Influence of ATRA on Hsp90-mediated tau protein uptake in HMO6 cells. **a**–**c** Assessment of tau protein absorption by HMO6 cells. **a** Cells were exposed to 1 µM ATRA for 24 h, followed by incubation with 10 ng/ml tau protein for various durations. **b** Cells were initially treated with varying concentrations of the Hsp90 inhibitor GeB for 2 h, then with 1 µM ATRA for 24 h, and finally with 10 ng/ml tau protein for 6 h. The intracellular accumulation of tau protein was quantified using flow cytometry and reported as tau protein uptake levels. **c** Cells were treated with 1 μM ATRA for 24 h followed by with or without 1 μM GeB for 2 h and then 6 h treatment with 10 ng/ml FITC-Tau protein. Cells were visualized using confocal microscope. (magnification ×200). *p < 0.05

Next, we sought to determine the role of cell surface Hsp90 in the internalization of tau protein in HMO6 cells. First, we investigate whether the presence of ATRA and tau protein could induce further expression of cell surface Hsp90. Interestingly, our result revealed that a low dose of tau (10 ng/mL) resulted in the induction of cell surface Hsp90 compared to only ATRA treatment (131.93%+/−8.13% compared to 116.92%+/−1.25%), whereas higher concentrations did not exhibit the same effect (Fig. S1). Consequently, we selected 10 ng/mL tau protein for subsequent experiments. To further explore the role of cell surface Hsp90 in tau uptake, cells were treated with 10 ng/ml of recombinant human tau proteins (hTau42) in the presence of 1 µM ATRA for different time points, and the level of intracellular tau protein was assessed by flow cytometry. As depicted in Fig. 2a, the 6 h tau-treated group showed a significantly increased intracellular tau protein levels (143.27%+/−7.64%), indicating the increased tau uptake. However, tau uptake levels decreased at 12 h and 24 h, suggesting possible tau release or degradation. To further explore the role of cell surface Hsp90 in tau uptake, we pre-treated cells with varying concentrations of cell surface Hsp90 inhibitor biotin-geldanamycin (GeB), prior to exposure to ATRA and tau protein. This inhibitor, coupled with biotin molecules, is a cell-impermeable agent, thereby specifically targeting the cell surface Hsp90 [12]. The intracellular level of tau protein significantly decreased after treatment with 1 µM (110.62%+/−3.69%) or 10 µM GeB (106.93%+/−6.5%) in the presence of ATRA compared to those without the inhibitor (127.61%+/−5.06%), as shown in Fig. 2b and c. This indicates that ATRA enhances the uptake of tau protein predominantly through cell surface Hsp90.

### Interaction Between Tau Protein and Hsp90 in HMO6 Cells

**Fig. 3:**
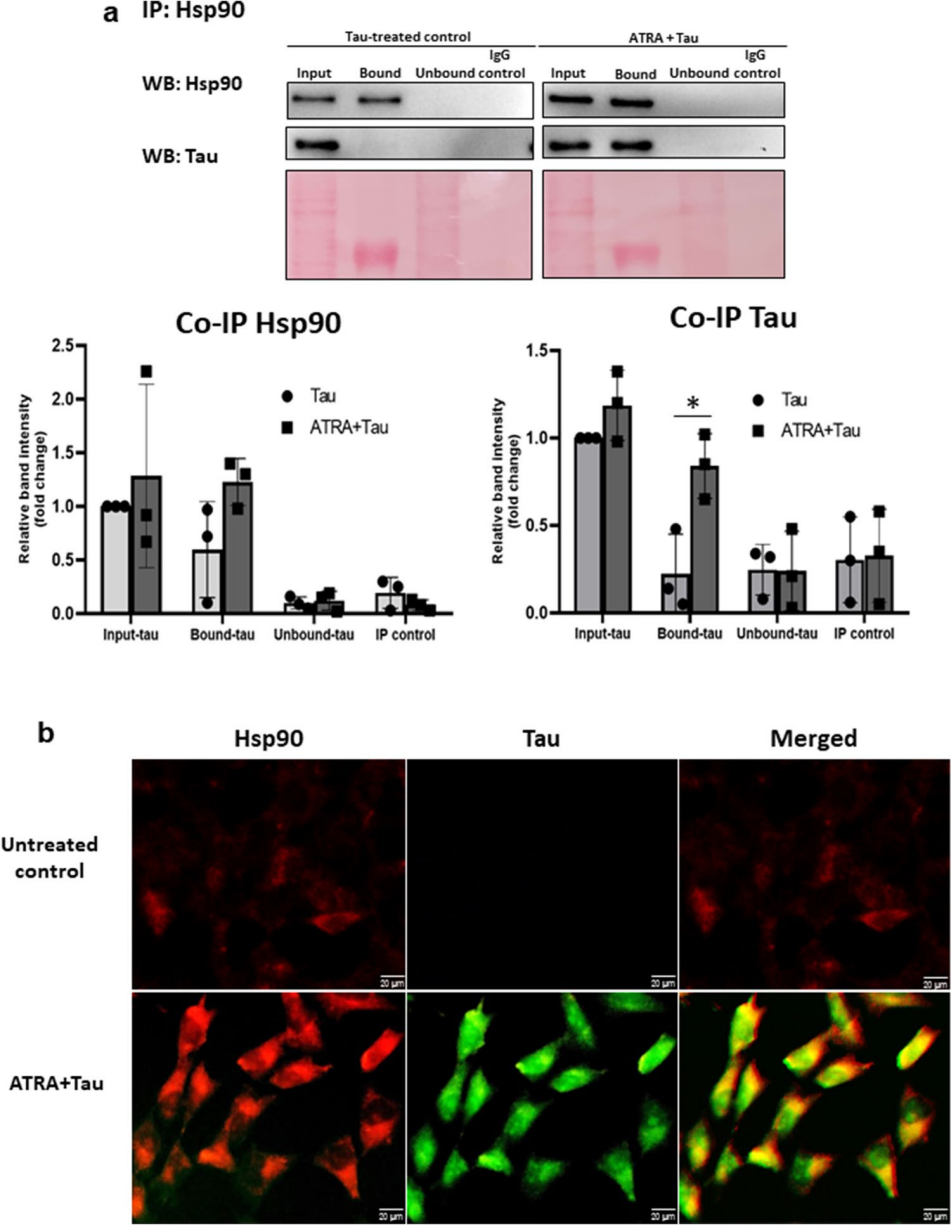
Interaction of tau proteins with Hsp90 in ATRA-treated HMO6 cells. **a** Hsp90 and tau protein co-immunoprecipitation. Cells were subjected to 1 µM ATRA for 24 h, followed by a 6-h exposure to 10 ng/ml tau protein. Subsequently, Hsp90 was immunoprecipitated from whole cell lysates using an anti-Hsp90 antibody and the association between Hsp90 and tau protein was analyzed via Western blot employing both anti-Hsp90 and anti-tau antibodies. IgG was used as a control antibody. Tau-treated group was used as the control. Densitometric analysis of the protein bands was performed using the ChemiDoc MP Imaging System (*n* = 3). **b** Co-localization study of Hsp90 and tau proteins. The cells were fixed in formaldehyde, permeabilized with Triton X-100, and incubated with anti-human tau antibody overnight at 4 °C. This was followed by a 2-h incubation with FITC-conjugated anti-human tau antibody (green) and subsequent incubation with Alexa 633-conjugated anti-Hsp90 antibody (red). The co-localization (yellow fluorescence) of Hsp90 and human tau protein was visualized using a fluorescence microscope at a magnification of ×200. *p < 0.05

To determine the direct interaction between Hsp90 and tau protein, cell lysates from ATRA+tau-treated cells underwent co-immunoprecipitation (Co-IP) using an anti-Hsp90 antibody. A clear band of tau protein detected in the Hsp90 Co-IP group indicated that tau protein was co-purified with Hsp90, as shown in Fig. 3a. To further confirm the interaction between Hsp90 and tau, a co-localization assay was employed. The merged image in Fig. 3b demonstrates the colocalization of the two proteins, indicated by the yellow color. These data suggest the binding of tau to Hsp90 to form an interacting complex.

**Fig. 4:**
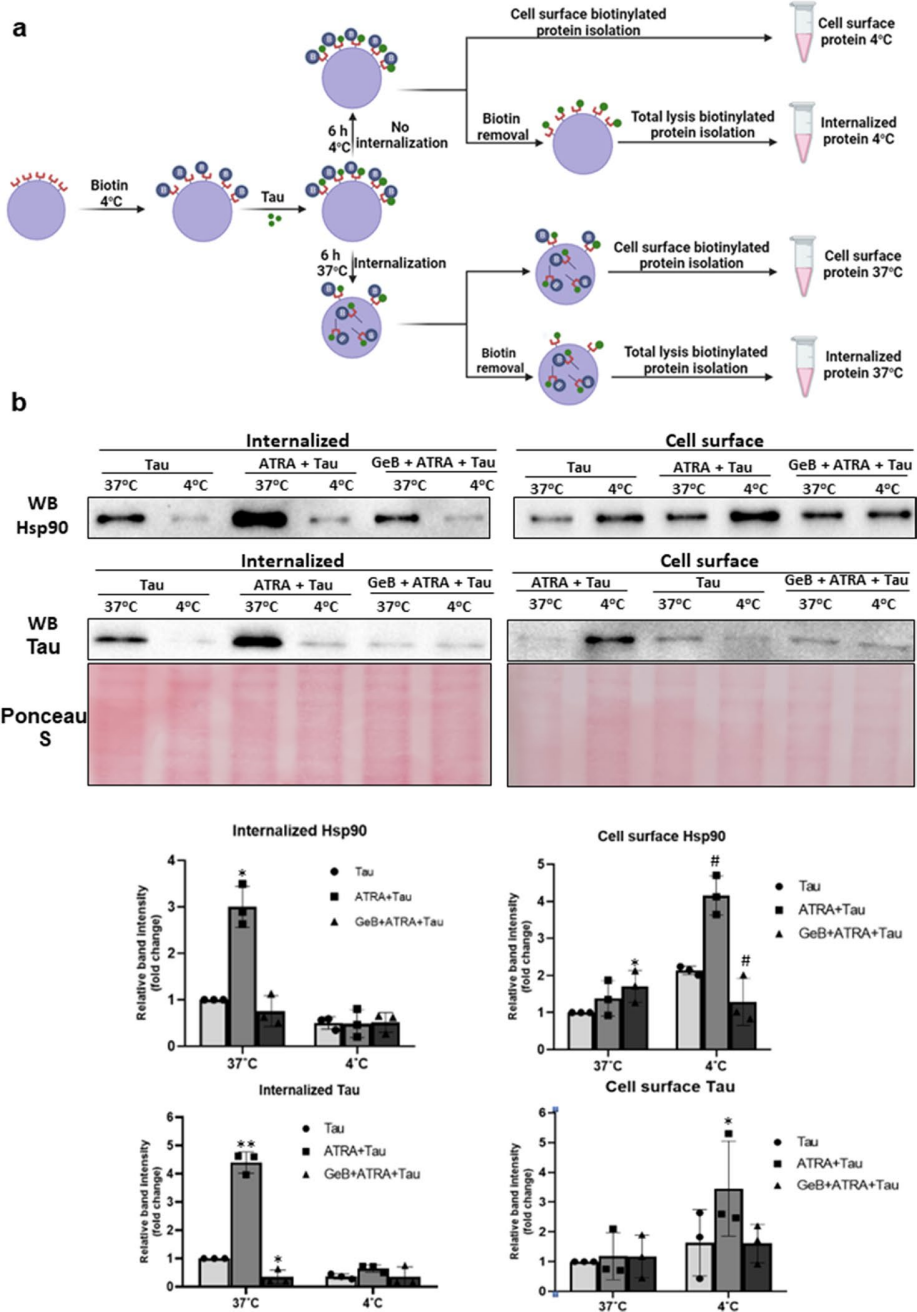

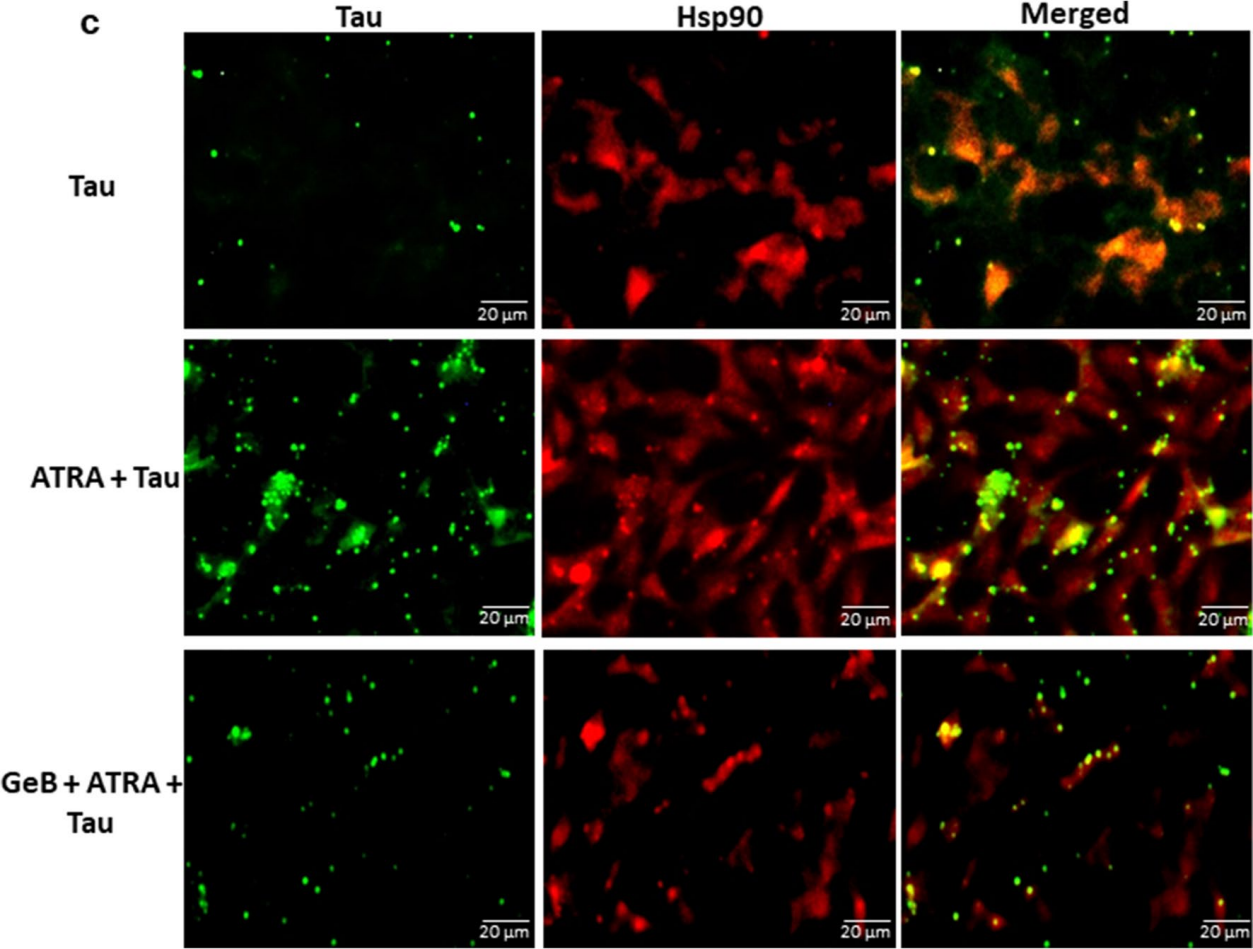
Tau internalization is mediated by cell surface Hsp90. **a** Schematic illustration of cell surface/internalized protein isolation by biotinylation assay. **b** After treatment with 1 µM ATRA or GeB + ATRA for 24 h, cells were labeled with 0.25 mg/mL Sulfo-NHS-SS-Biotin for 30 min at 4 °C followed by treatment with 10 ng/mL of human recombinant tau protein and incubated at 4 °C or 37 °C for 6 h. The cell surface or internalized biotinylated protein was isolated using the avidin column and subjected to the western blot analysis with the antibodies against Hsp90 and human tau. Tau-treated group was used as the control. Densitometric analysis of the protein bands was performed using the ChemiDoc MP Imaging System (*n* = 3). **c** After treatment time, the cells were fixed in formaldehyde, permeabilized with Triton X-100, and incubated with anti-human tau antibody overnight at 4 °C. This was followed by a 2-h incubation with FITC-conjugated anti-human tau antibody (green) and subsequent incubation with Alexa 633-conjugated anti-Hsp90 antibody (red). The co-localization (yellow fluorescence) of Hsp90 and human tau protein was visualized using a fluorescence microscope at a magnification of ×200. *p < 0.05 vs tau 37 °C group; #*p <* 0.05 vs tau 4 °C group

For further elucidate the role of Hsp90 in tau internalization, we assessed the Hsp90 and tau level on cell surface and intracellularly under normal condition and conditions where internalization was blocked (Fig. 4a). As shown in Fig. 4b, under normal conditions, both intracellular Hsp90 and tau were induced, while the cell surface expression was decreased. However, incubation at 4 °C resulted in a decrease in the intracellular level of both proteins, indicating co-internalization of Hsp90 and tau under ATRA treatment. In the presence of GeB, the intracellular but not cell surface level of Hsp90 was reduced compared to ATRA-treated group, suggesting that GeB inhibits the function without affecting its expression level. GeB treatment also led to a reduction of intracellular tau, indicating the involvement of Hsp90 in tau internalization. This finding was further supported by fluorescence microscopy experiment, which revealed a decrease in intracellular tau protein (green) in the presence of GeB compared to the ATRA-treated group (Fig. 4c).

### ATRA Facilitates Tau Uptake Via Caveolae/Raft-Dependent Endocytosis

**Fig. 5:**
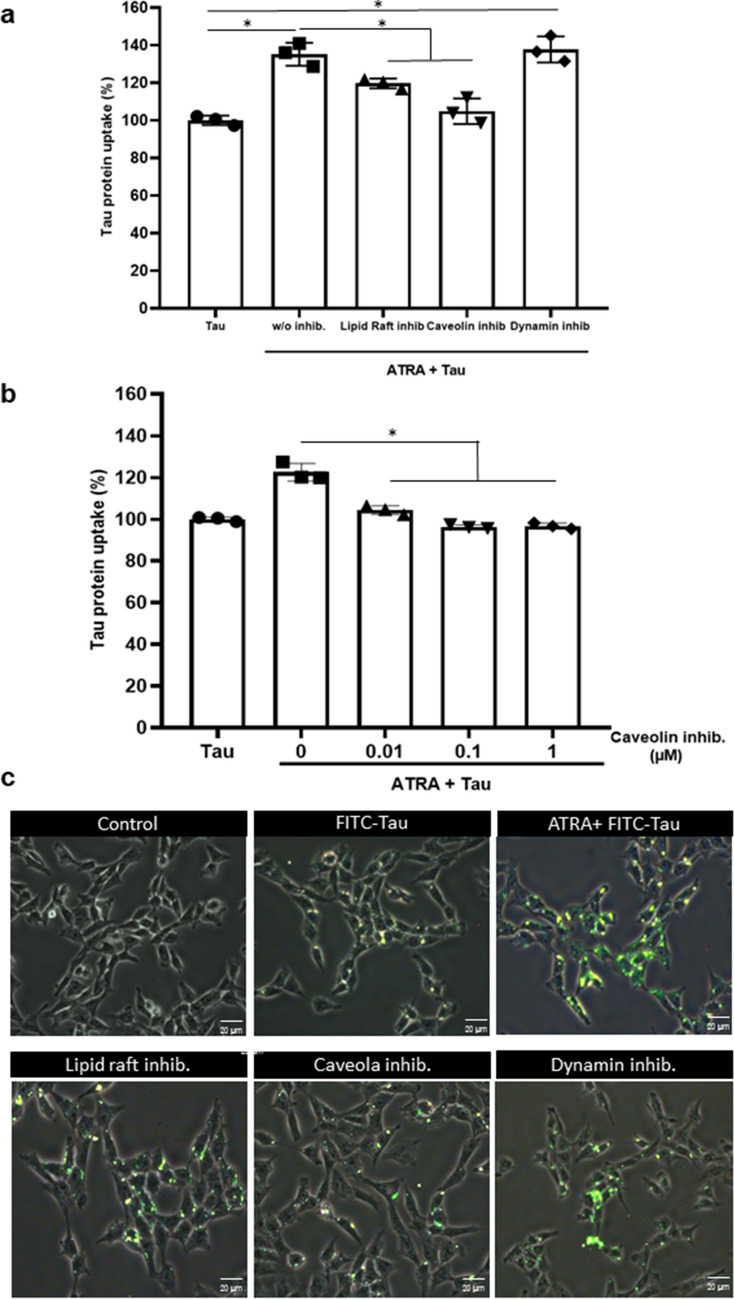
ATRA-enhanced tau protein uptake in HMO6 cells via caveolae/raft-dependent endocytosis. **a**–**c** Evaluation of tau protein absorption in the context of endocytosis inhibition. HMO6 cells were treated with 1 µM ATRA for 24 h and subsequently exposed to various endocytosis inhibitors: 5 mM methyl-β-cyclodextrin (lipid raft inhibitor), 0.1 µM filipin III (caveolin inhibitor), and 10 µM Dyngo® 4a (dynamin inhibitor) for 2 h (**a**). This was followed by different concentrations of the caveola-dependent endocytosis inhibitor Filipin III (**b**). After these treatments, cells were incubated with tau protein for 6 h. The cells were then harvested, and intracellular tau protein levels were quantified using flow cytometry (**a**, **b**) and fluorescence microscopy (magnification ×200) (**c**). *p < 0.05

To investigate the pathway involved in the internalization of tau by microglia under ATRA treatment, we utilized various endocytosis inhibitors, including Methyl-β-cyclodextrin (a specific inhibitor for lipid raft-mediated endocytosis) and Filipin III (a specific inhibitor for caveolae-mediated endocytosis) as clathrin-independent endocytosis inhibitors, and Dyngo® 4a as a clathrin-dependent endocytosis inhibitor. As shown in Fig. 5a, inhibitors of clathrin-independent endocytosis significantly suppressed the level of intracellular tau protein (119.63%+/−6.78% and 104.8%+/−6.78%), whereas the clathrin-dependent endocytosis inhibitor did not affect tau uptake (137.71%+/−6.9%). Notably, lower concentrations of caveolin inhibitor (0.01 µM filipin III) markedly inhibited tau uptake (104.36%+/−2.11%), suggesting a predominant role of caveolae-mediated endocytosis (Fig. 5b). Consistent with this result, immunofluorescence data showed a reduction in the internalized tau signal in the presence of clathrin-independent endocytosis inhibitors (Fig. 5c). These results suggest that tau is primarily internalized through caveolae/raft-dependent pathways.

### ATRA Enhances Tau Degradation Via Lysosomal and Proteasomal Pathways

**Fig. 6:**
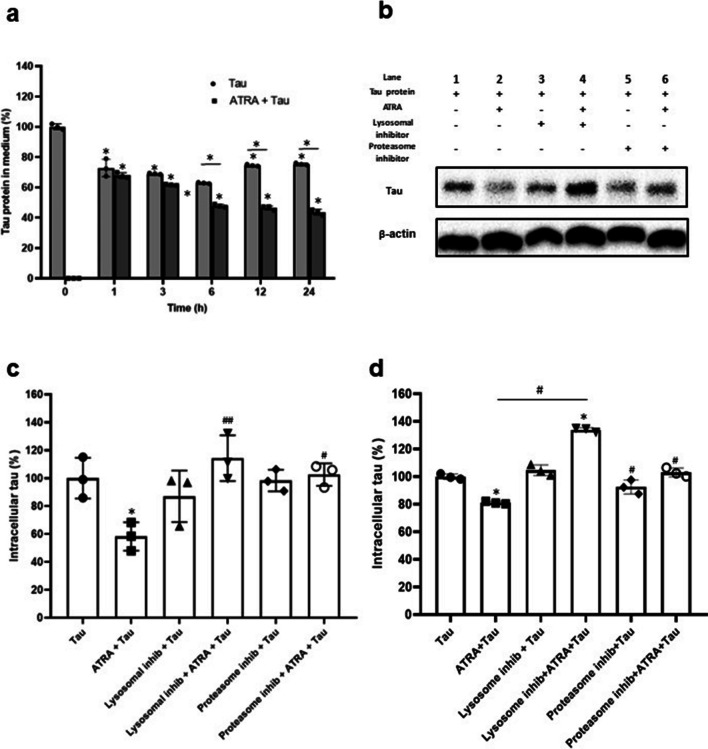
Degradation of ATRA-enhanced intracellular tau proteins in HMO6 cells via lysosomal and proteasomal pathways. **a** Measurement of tau protein in culture medium post-treatment. Following 24 h of 1 µM ATRA treatment, cells were incubated with 10 ng/ml tau protein for varying periods. Subsequently, the culture medium was collected, and tau protein levels were determined using ELISA. The baseline (0 h tau levels) represents the tau protein concentration immediately after the addition of tau protein. **b**–**d** Analysis of intracellular tau protein levels in the context of protease inhibition. Cells were treated with or without ATRA, in the presence of either lysosomal inhibitor (leupeptin, 50 µM) or proteasome inhibitor (epoxomicin, 1 nM) for 24 h, followed by 10 ng/ml tau protein treatment for another 24 h. The cells were then harvested, and intracellular tau protein was detected via Western blot (**b**). Densitometric analysis of the tau protein bands was performed using the ImageJ software (**c**). In parallel experiments, cells were treated with lysosomal (Leupeptin) or proteasome (Epoxomicin) inhibitors combined with ATRA for 24 h, followed by 10 ng/ml tau protein for an additional 24 h. Intracellular tau protein levels were then quantified using flow cytometry (**d**). *p < 0.05 compared to 0 h Tau (**a**), *p < 0.05 compared to Tau only (**c**, **d**), #*p <* 0.05 and ##*p <* 0.01 compared to ATRA+Tau (**c**, **d**)

After internalization, tau protein can be degraded via two major systems: the autophagy-lysosomal and the ubiquitin-proteasome pathways. Alternatively, tau can be secreted into the extracellular space. The flow cytometry results shown in Fig. 2a indicated that tau uptake was significantly induced after 6 h of treatment with tau but was dramatically reduced at longer incubation times (12 h and 24 h). To understand the observed drop in internalized tau levels, we first measured the extracellular level of tau in the medium using indirect ELISA. Figure 6a shows that after 6 h, 12 h, and 24 h of tau protein treatment, tau release decreased rapidly in the presence of ATRA compared to the non-treated group (47.8%+/−1.03%; 46.43%+/−1.42%; 43.56%+/−1.88% compared to 100%+/−1.85%). Conversely, in the group treated with tau alone, the levels of tau in the medium increased after 12h and 24h (74.52%+/−0.62% and 75.34%+/−0.62%), suggesting the release of intact tau in the absence of ATRA. To determine which system regulates tau degradation in ATRA-treated microglia, we evaluated tau levels in both the extracellular environment and the intracellular matrix. Leupeptin and epoxomicin were used as lysosomal and proteasome inhibitors, respectively. Using Western blot and flow cytometry, we assessed the intracellular tau in the presence of these treatments. Figure 6b–d demonstrates that ATRA-induced tau degradation was inhibited under treatment with either lysosomal or proteasome inhibitors. This indicates that ATRA facilitates tau degradation through both lysosomal and proteasomal pathways. In contrast, tau levels in non-ATRA-treated cells remained unaffected by these inhibitors, suggesting that in the absence of ATRA, majority of tau is secreted rather than undergoing intracellular processing and degradation.

## Discussion

**Fig. 7:**
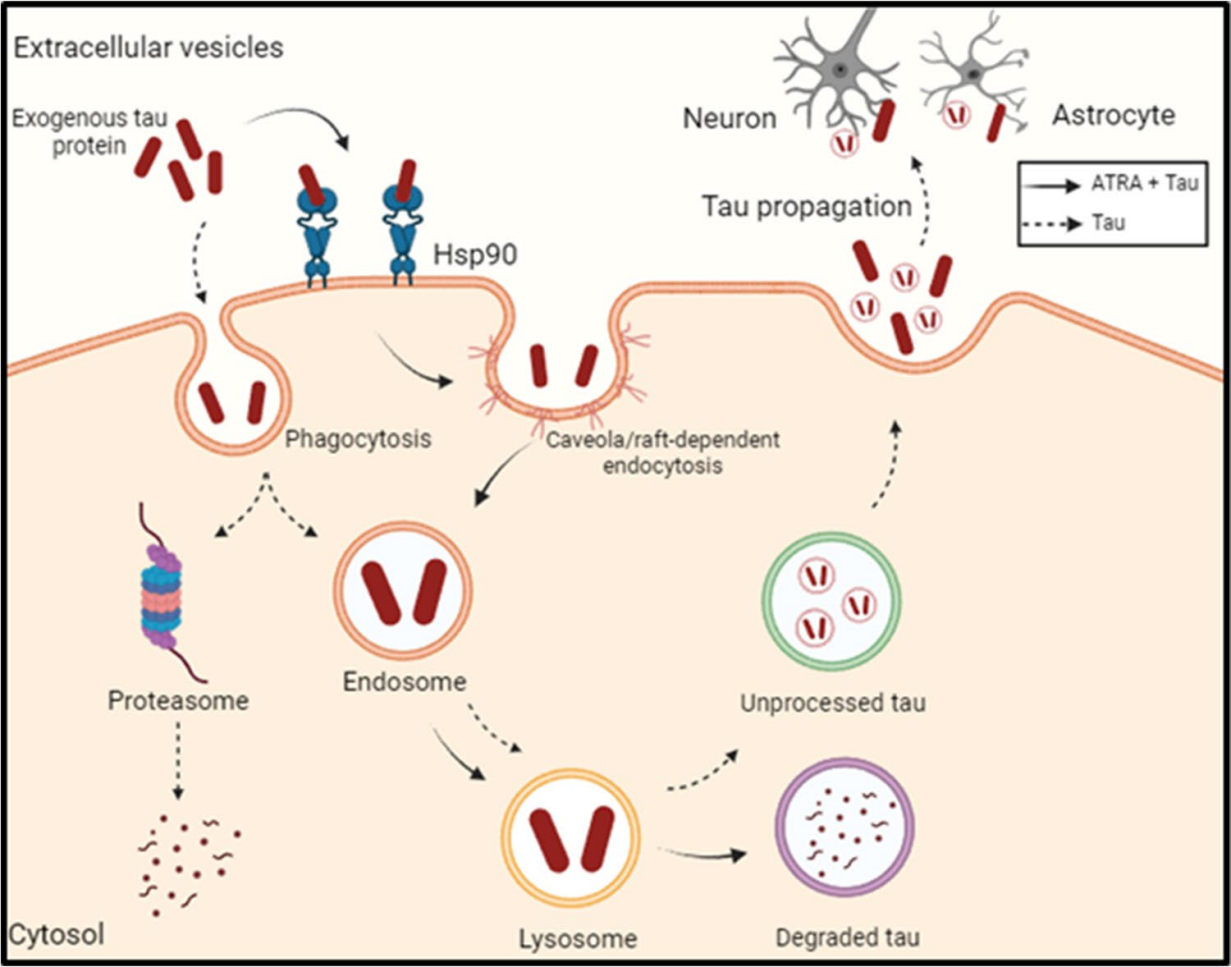
Schematic overview of Hsp90-facilitated tau protein uptake and degradation in ATRA-treated human microglial cells. The diagram illustrates the upregulation of cell surface Hsp90 following ATRA treatment, which subsequently binds to exogenous tau proteins. This interaction promotes tau protein uptake into the microglia via clathrin-independent endocytosis, leading to intracellular routing of tau proteins to endosomes and lysosomes. Inside lysosomes, the tau proteins undergo degradation, while some remain unprocessed. The schematic also depicts tau propagation between neurons and astrocytes, highlighting the differences in tau processing in the presence and absence of ATRA. Solid lines indicate pathways influenced by ATRA plus tau treatment, and dashed lines represent the tau pathway in the absence of ATRA

In this study, we have demonstrated that ATRA enhances Hsp90 expression on the surface of human microglia, which in turn mediates tau uptake via caveolae/lipid raft endocytosis pathways and facilitates tau degradation through lysosomal and proteasomal pathways (Fig. 7). This finding aligns with previous research suggesting the role of extracellular Hsp90 as a transporter for various proteins [10, 21–23]. Furthermore, our results add to the existing body of literature on the interaction between extracellular tau and Hsp90, showing an interaction between exogenous tau and Hsp90, and emphasizing Hsp90’s crucial role in tau uptake, as demonstrated by the inhibition of tau uptake by microglia using an Hsp90-selective inhibitor [8, 24–27].

Hsp90 acts as a key mediator of cellular homeostasis and serves as a molecular chaperone for various oncoproteins implicated in cancer development. Overexpression of Hsp90 aids tumor cell survival in harsh environments by stabilizing these oncoproteins [28]. Conversely, ATRA exerts its anti-cancer effects by activating retinoic acid receptors, which subsequently influence numerous genes involved in cell cycle arrest, differentiation, and apoptosis in cancer cells [29]. In our study, we observed that ATRA initially induced cell surface Hsp90 expression, which subsequently declines over time. Conversely, intracellular Hsp90 levels decrease during early ATRA treatment but exhibit an increase over time. These findings suggest that ATRA treatment in microglial cells may modulate Hsp90 localization rather than enhancing its overexpression. This modulation likely plays a role in regulating Hsp90 activity, potentially contributing to cellular defense mechanisms against pathological conditions. However, the results obtained from our biotinylation assay showed that in the presence of Hsp90 impermeable inhibitor GeB, both intracellular and cell surface Hsp90 levels were decreased under conditions where internalization was blocked (4 °C). This could be attributed to the inhibitor’s effect on the intracellular trafficking or recycling pathways of Hsp90, as suggested by Hartl’s group [30]. Further in-depth studies will be required to unravel the underlying mechanisms driving this observed phenomenon.

Fibrillary and aggregated tau proteins, formed by hyperphosphorylated tau, are widely recognized as major contributors to tauopathies [31]. However, emerging evidence suggests that monomeric tau, previously considered benign in the brain, may play a role in tau toxicity and propagation [32–35]. Although endogenous tau is not typically expressed in microglia [36, 37], intracellular tau protein can be detected and may contribute to the spread of tau toxicity through the internalization of exogenous tau from extracellular fluid. Monomeric tau activates the PQBP1-cGAS-STING pathway in microglia, promoting brain inflammation [38], and can trigger the microglial NF-κB mechanism, driving tau propagation in mouse models of tauopathy [39]. Thus, soluble forms of tau, such as monomeric tau protein, warrant greater attention to prevent tau proliferation, especially in the early stages of disease. Therefore, our study focuses on elucidating the potential role of microglia in clearing exogenous monomeric tau, offering a new approach to mitigating toxicity associated with tau pathology. Our findings revealed an interesting observation that at lower tau concentrations, the expression level of cell surface Hsp90 was induced compared to the higher concentrations. This implies that lower doses of tau may more effectively enhance the response of cell surface Hsp90 more effectively compared to higher concentrations, suggesting a potential cellular protective effect of Hsp90 in response to low doses of the pathological agents.

Our study uniquely contributes to the understanding of tau protein internalization via caveolae/lipid raft endocytosis pathways in microglia, contrasting with the clathrin-dependent pathway observed in neurons [40–43]. This suggests potential specificity in therapeutic targeting for disorders characterized by tau accumulation. Moreover, the finding that microglia degrade internalized tau proteins through ubiquitin-proteasome and autophagy-lysosome systems, including chaperone-mediated autophagy (CMA), highlights the importance of efficient protein degradation mechanisms in neurodegenerative diseases [36, 44, 45].

Role of ATRA in enhancing surface Hsp90 expression in microglia, previously observed in human macrophages, suggests a broader implication for ATRA in cellular signaling and protein degradation, particularly relevant in the context of neurodegenerative diseases [10]. This enhancement of microglial function could contribute to the mitigation of neurodegeneration in conditions such as Alzheimer’s disease and other tauopathies, as supported by previous studies [46–48].

The broader implications of our findings extend to the role of vitamin A in neurodegeneration. Vitamin A deficiency is associated with cognitive impairments, and activation of RAR and RXR in AD models has been shown to improve symptoms, including reduced Ab deposition and tau hyperphosphorylation [49–53]. Furthermore, anti-inflammatory properties of ATRA, which downregulate pro-inflammatory cytokines and chemokines in astrocytes and microglia, underscore its potential in neuroprotection [54].

However, our study is not without limitations. Firstly, given the in vitro nature of our research, it is imperative to validate these findings in vivo to better understand the molecular mechanisms underlying ATRA-induced changes in microglia and to assess ATRA’s therapeutic potential in clinical settings. Additionally, a comprehensive exploration of ATRA’s impact on various CNS cell types, including neurons, astrocytes, and oligodendrocytes, is warranted. Secondly, while our study concentrated on a single isoform of monomeric tau protein, future investigations should expand to include a spectrum of tau protein forms, ranging from monomers to oligomers and aggregated variants. Such studies could offer more profound insights into the pathophysiology of neurodegenerative diseases and inform therapeutic development. Thirdly, elucidating the broader biological roles of Hsp90 in protein folding and degradation is essential for advancing the development of effective treatments for Alzheimer’s disease and related neurodegenerative disorders. Finally, considering the known limitations of ATRA in clinical application [55, 56], as well as issues related to poor solubility and bioavailability, it is important to perform in-depth investigations on optimizing ATRA delivery methods and exploring synergistic combination therapies.

In conclusion, our findings suggest potential therapeutic benefits of ATRA in Alzheimer’s disease, highlighting the importance of further research into tau internalization and degradation mechanisms. This could pave the way for developing more effective treatments for Alzheimer’s disease and related neurodegenerative disorders.

## Supplementary Information

Below is the link to the electronic supplementary material.

Supplementary file1 (DOCX 1066 KB)

## Data Availability

The datasets generated and/or analyzed during the current study are available from the corresponding author upon reasonable request.

## Abbreviations

ATRA: all-trans retinoic acid
Hsp90: heat shock protein 90
AD: Alzheimer’s disease
