# Supplementary material for: All-Trans Retinoic Acid-Induced Cell Surface Heat Shock Protein 90 Mediates Tau Protein Internalization and Degradation in Human Microglia
Source: Mol Neurobiol. 2024 Jun 20;62(1):742–55. doi: 10.1007/s12035-024-04295-1 (PMC11711573; doi:10.1007/s12035-024-04295-1)
Supplement: Supplementary file 1 — Supplementary file1 (DOCX 1066 KB) [file 12035_2024_4295_MOESM1_ESM.docx]

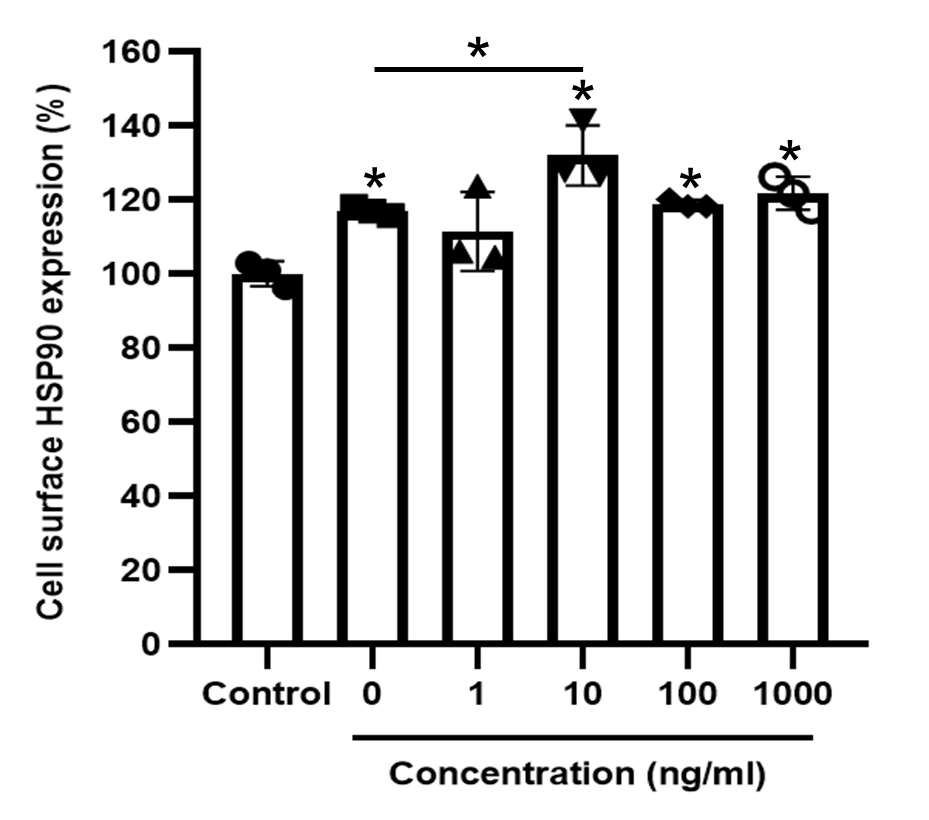
Supplementary Material

**Supplementary Figure 1. Tau treatment induces cell surface Hsp90 expression.** Cells were treated with 1 µM ATRA for 24 h followed by various concentrations of human Tau protein for 24 h. Cell surface Hsp90 expression was measured by flow cytometry. HMO6 cell non-treated with ATRA or tau was used as a control group.* p < 0.05 vs control.


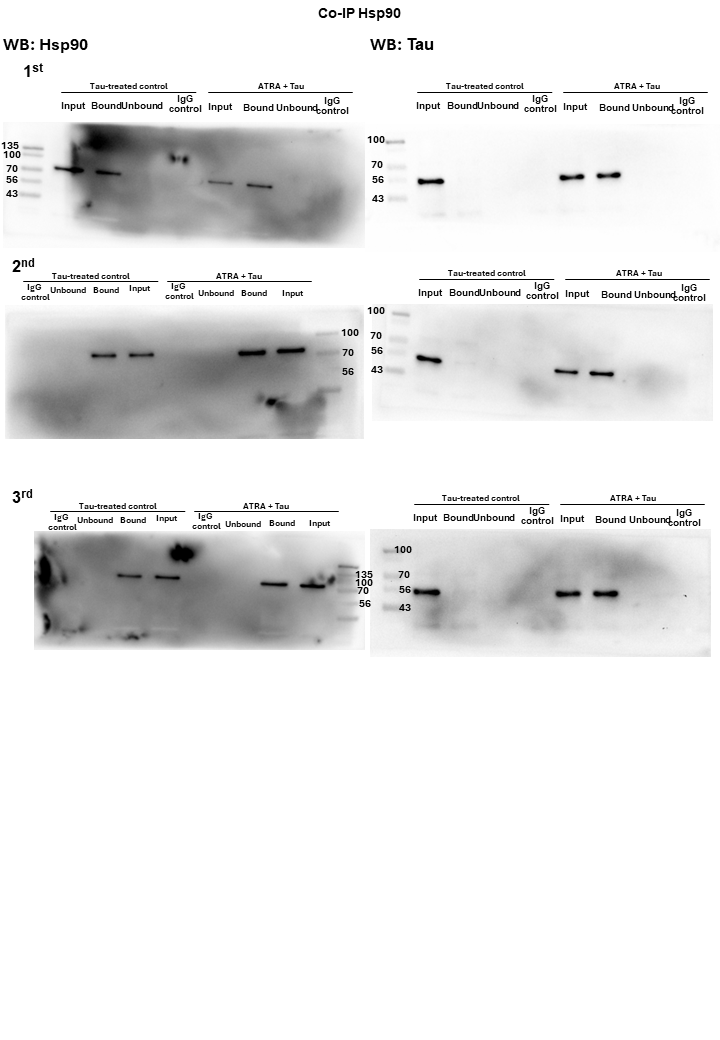


**Supplementary Figure 2. Original blots of Fig. 3A-Interaction of tau proteins with Hsp90 in ATRA-treated HMO6 cells.** Hsp90 and tau protein co-immunoprecipitation. Cells were subjected to 1 µM ATRA for 24 h, followed by a 6-h exposure to 10 ng/ml tau protein. Subsequently, Hsp90 was immunoprecipitated from whole cell lysates using an anti-Hsp90 antibody and the association between Hsp90 and tau protein was analyzed via Western blot employing both anti-Hsp90 (A) and anti-tau antibodies (B). IgG was used as a control antibody. (n=3).


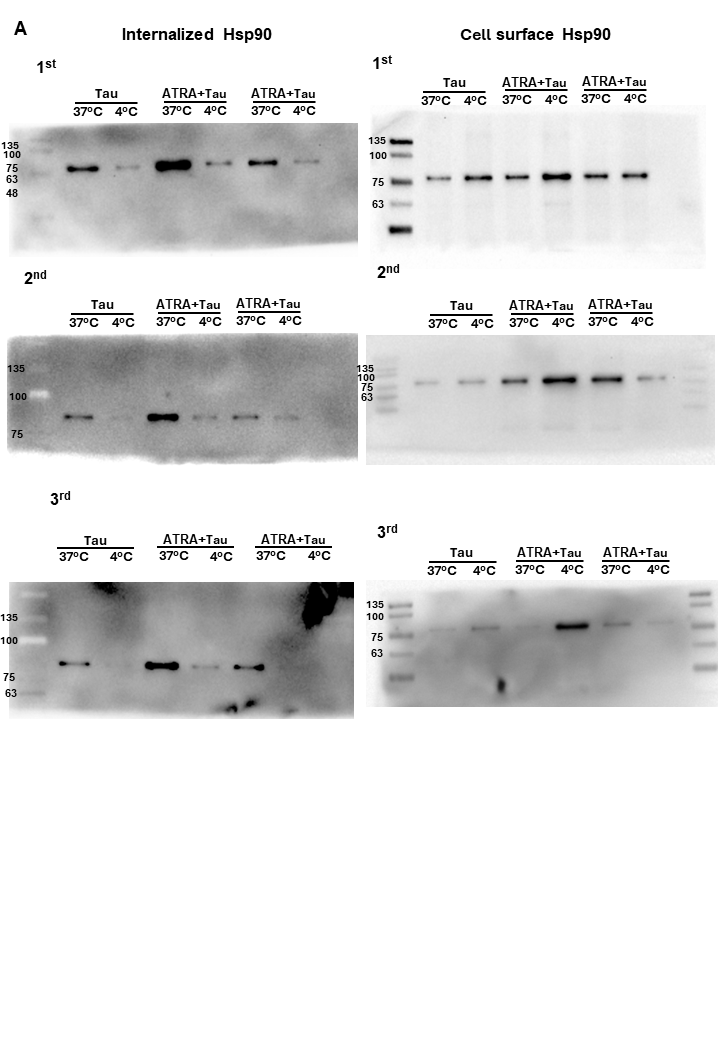


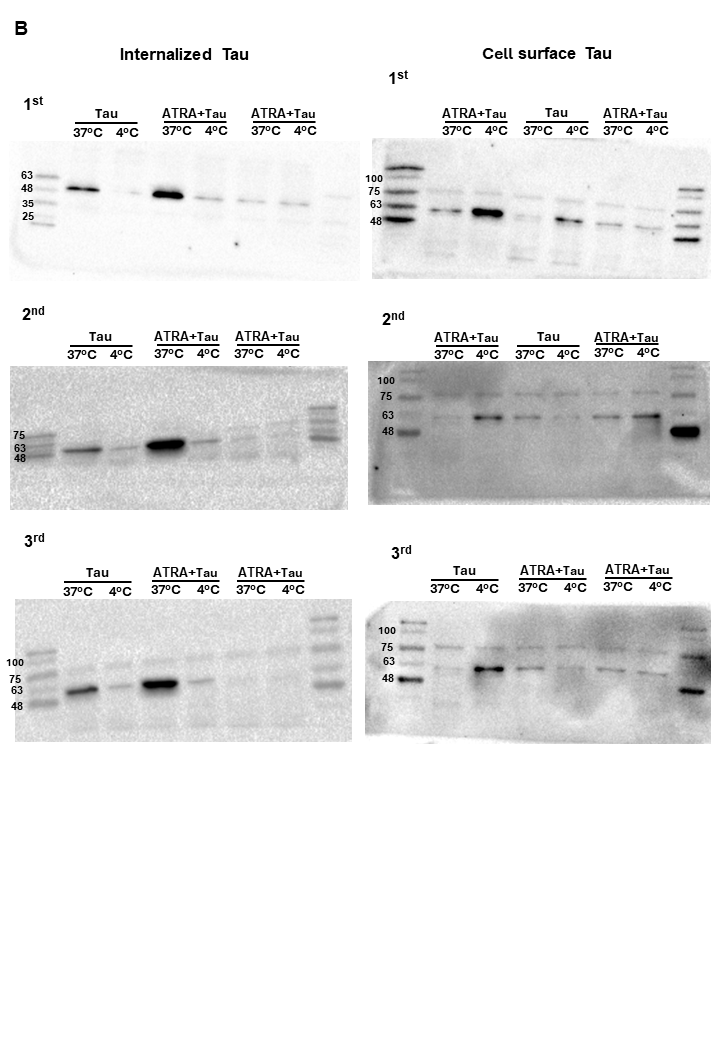


**Supplementary Figure 3. Original blots of Fig. 4B-Tau internalization is mediated by cell surface Hsp90**. After treatment with 1 µM ATRA or GeB + ATRA for 24 h, cells were labeled with 0.25 mg/mL Sulfo-NHS-SS-Biotin for 30 min at 4 ^o^C followed by treatment with 10 ng/mL of human recombinant tau protein and incubated at 4 ^o^C or 37 ^o^C for 6 h. The cell surface or internalized biotinylated protein was isolated using the avidin column and subjected to the western blot analysis with the antibodies against Hsp90 (A) and human tau (B). (n=3).


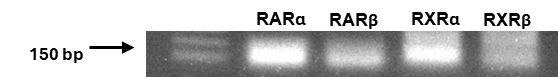


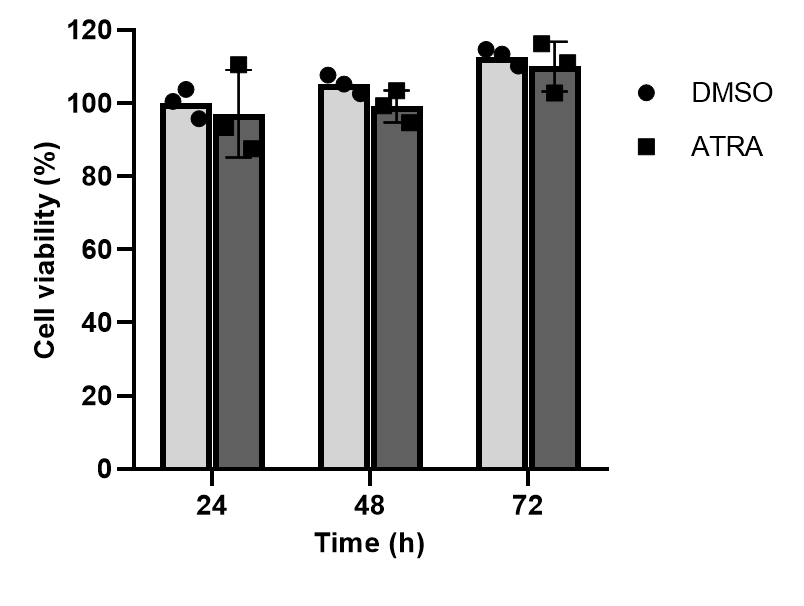
 **Supplementary Figure 4. Expression level of retinoic acid receptor encoding genes in human microglial cell HMO6**. Total RNA from HMO6 cell was extracted using the easy-BLUETM Total RNA Extraction Kit and quantified with a spectrophotometer. cDNA was synthesized from 2 µg of total RNA. Genes encoding for RARα, RARβ, RXRα, RXRβ were amplified using RT-PCR. The PCR products were run on a 1 % agarose gel and the image was visualized using a ChemiDoc MP machine. Primers used were RARα 5′-GAAGTGCTTTGAAGTGGGCA-3′ and 5′-CCGTAGTGTATTTGCCCAGC-3′; RARβ 5′-GCAAGCCTCACATGTTTCCA-3′ and 5′-TGACCCCACTGTTTTCCACT-3′; RXRα 5′-ATACTCTTGCCGGGACAACA-3′ and 5′-TCCCCATCCTTGTCCTTTCC-3′; RXRβ 5′-ATACTCTTGCCGGGACAACA-3′ and 5′-TCCCCATCCTTGTCCTTTCC -3′

**Supplementary Figure 5. ATRA treatment does not affect the cell viability of HMO6 cell line in different timepoints**. Cells were treated with DMSO or 1 µM ATRA for 24, 48 and 72 h. Cell viability was determined by the EZ-Cytox assay. ns: non-significance compared to DMSO 24 h group.

**Table S1**. Putative binding site of transcription factors on *Hsp90α* promoter region.

| **Transcription factor** | **Start position** | **End position** |
| --- | --- | --- |
| **RXRα** | 69 | 75 |
| **RXRα** | 75 | 81 |
| **RXRα** | 599 | 605 |
| **RARβ** | 67 | 76 |
